# Piceatannol, a Natural Polyphenol From Grape, Inhibits *Helicobacter pylori* Through Targeted Suppression of Its Secreted Urease

**DOI:** 10.1002/fsn3.70932

**Published:** 2025-09-08

**Authors:** Qiang Lu, Yuhong Xie, Xuefei Wang, Haohui Chen, Yifei Xu, Cailan Li

**Affiliations:** ^1^ Department of Pharmaceutical Sciences Zunyi Medical University Zhuhai People's Republic of China; ^2^ Department of Pharmacology Zunyi Medical University Zhuhai People's Republic of China; ^3^ Shenzhen Traditional Chinese Medicine Hospital The Fourth Clinical Medical College of Guangzhou University of Chinese Medicine Shenzhen People's Republic of China

**Keywords:** enzyme kinetics, Helicobacter pylori, piceatannol, polyphenol, thiol group, urease

## Abstract

*Helicobacter pylori* infection, mediated largely through its urease enzyme, causes chronic gastric inflammation that can progress to severe pathology. This study investigates the inhibitory effects of piceatannol (PIC), a polyphenol from grape peels, on *H. pylori* growth and its urease activity. PIC remarkably inhibited the growth of three *H. pylori* strains, with MIC values ranging from 100 to 150 μg/mL. Furthermore, PIC exerted a potent inhibitory effect on *H. pylori* urease (HPU) and jack bean urease (JBU) with IC_50_ values of 0.67 ± 0.02 mM and 0.47 ± 0.01 mM, respectively. Kinetic studies revealed that PIC acts as a slow‐binding, mixed inhibitor of HPU and a slow‐binding, non‐competitive inhibitor for JBU. The use of thiol‐blocking substances observably delayed the deactivation of HPU and JBU, whereas the synergistic effect of PIC with competitive inhibitors of Ni^2+^ further restrained urease activity. These results indicated that the sulfhydryl group in the active site may be the key binding site for PIC to inhibit HPU and JBU. Molecular docking simulations further support the inhibitory mechanism. Additionally, the inactivation of HPU and JBU caused by PIC was reversible. In conclusion, PIC significantly suppresses the growth of *H. pylori* and the activity of its major virulence factor, urease. Further investigation into the kinetic properties and mechanisms revealed that the inhibition of urease by PIC may target the active site sulfhydryl group.

## Graphical abstract

Piceatannol significantly inhibits the growth of *Helicobacter pylori* through targeted suppression of its secreted urease activity. The inhibition of piceatannol against *H. pylori* urease (HPU) and jack bean urease (JBU) was characterized in a slow‐binding, mixed‐type and slow‐binding, noncompetitive type, respectively. The inhibition mechanism of HPU and JBU by piceatannol was demonstrated to involve the blockage of the sulfhydryl group at the active site of the enzyme.

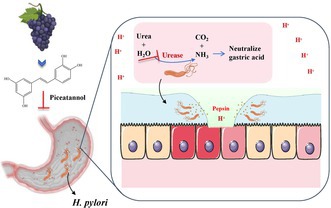

## 1 Introduction

*Helicobacter pylori* ( *H. pylori* ) is a Gram‐negative bacterium that resides in the gastrointestinal tract. This pathogen is a common cause of various gastrointestinal disorders, including gastritis, peptic ulcers, and gastric cancer (Zhao et al. 2024). With over 50% of the global population infected, *H. pylori* poses a significant public health burden, particularly in developing countries where prevalence rates exceed 80% (Sharara et al. 2025). Typically, the treatment for *H. pylori* infections involves a combination of antibiotics and complementary medications (Ford et al. 2022). However, the extensive use of these antimicrobials presents significant challenges, such as adverse effects and the emergence of antibiotic resistance (Medakina et al. 2023). The rising prevalence of multidrug‐resistant *H. pylori* strains has rendered standard therapies ineffective in up to 30% of cases, underscoring the urgent need for novel therapeutic strategies (Salahi‐Niri et al. 2024). Novel approaches, including bioengineered soft robots for targeted drug delivery (Wang, Chen, et al. 2024) and the use of natural compounds, are being explored to overcome these limitations.

Urease (EC 3.5.1.5) is an enzyme rich in thiols that catalyzes the hydrolysis of urea to ammonia and carbamate. It is derived from various sources comprising plants, bacteria, fungi, and invertebrates (Ray et al. 2020). The urease synthesized by jack bean ( *Canavalia ensiformis* ) seeds was the first metalloenzyme discovered to contain nickel ions, and it is commonly used in the research of urease inhibitors (Kappaun et al. 2018). The active site of urease consists of sulfhydryl groups, two Ni^2+^ ions, and a carboxylated lysine that acts as a bridging bond (Mazzei et al. 2020). Among these components, the Ni^2+^ ions and the sulfhydryl groups, particularly the multifarious cysteine residues in the enzyme's active site, exert a vital function in the catalytic activity of all enzymes (Li et al. 2018).

Numerous research studies indicate that urease represents a considerable threat in various fields, including medicine, agriculture, and animal husbandry (He et al. 2022). In the field of medicine, bacterial urease serves as a key virulence factor in various conditions, the most important of which is *H. pylori* urease (HPU). HPU is primarily synthesized in the cytoplasm and subsequently localized to both the bacterial surface and extracellular milieu through secretory pathways (Mobley et al. 1995). This dual localization enables its pH‐neutralizing function in the gastric environment. HPU is a cytoplasmic protein present on bacterial surfaces and is a Ni^2+^‐containing metalloenzyme released by *H. pylori* , which was recognized as a significant colonization and pathogenic factor of *H. pylori* (Eftekhari et al. 2021). It decomposes urea into ammonia, which neutralizes stomach acid and generates a nearly neutral environment. This enables *H. pylori* to survive in the stomach, promoting inflammation and growth, eventually causing duodenal and peptic ulcers as well as gastric cancer (Xia et al. 2024). Hence, blocking urease is a key step in treating infections caused by bacteria. In agriculture, urease present in soil can rapidly hydrolyze urea, producing a large amount of ammonia, which may lead to an increase in soil pH (Krol et al. 2020). Crops are unable to fully absorb this ammonia in a short period of time, leading to unproductive volatilization and reduced utilization efficiency of urea, resulting in low economic returns (Afshar et al. 2018; Rodriguez et al. 2021). Therefore, research on urease inhibitors can effectively inhibit the decomposition of urea and reduce the release of ammonia, which is an important measure to address medical and agricultural concerns.

Currently, the urease inhibitors that have been studied and used by domestic and foreign researchers are mainly hydroxamic acids, heavy metal ions, phosphate esters, and imidazole derivatives (Song, Liu, Li, and Xiao 2022; Song, Liu, Yuan, et al. 2022; Zhao et al. 2021; Mazzei, Cianci, Benini, and Ciurli 2019; Nain‐Perez et al. 2019; Cheng et al. 2019). However, most existing inhibitors are limited in practical use due to poor stability, apparent toxic side effects, and high cost (Song, Liu, Li, and Xiao 2022; Song, Liu, Yuan, et al. 2022). Thus, there is an urgent need to develop urease inhibitors with fewer side effects, higher bioactive properties, and low cost.

**FIGURE 1:**
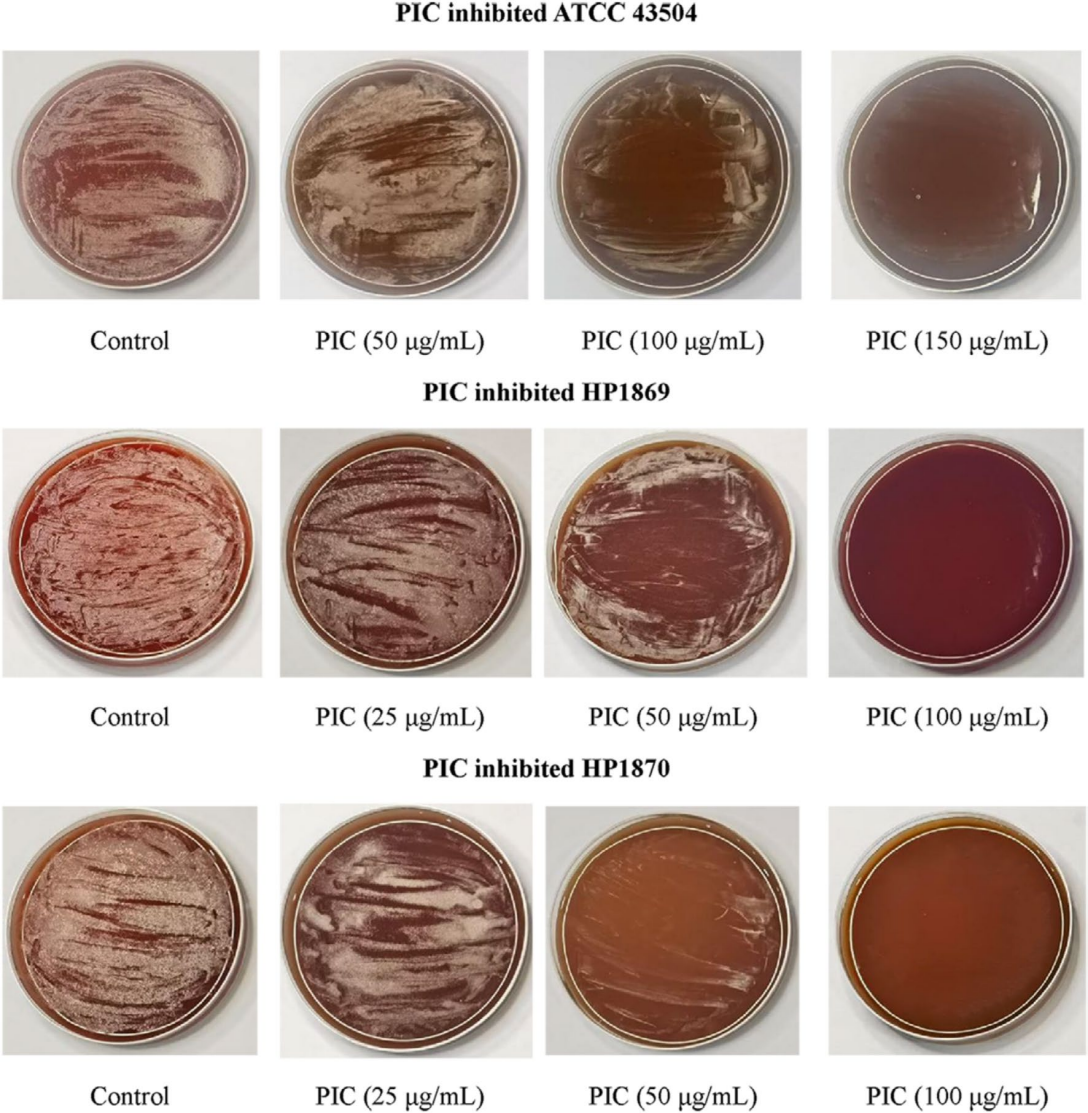
Determination of the minimal inhibitory concentrations (MICs) of Piceatannol (PIC) for various strains of *Helicobacter pylori* .

Piceatannol (PIC, C_14_H_12_O_4_, Figure 1) is a polyphenolic compound, as a derivative of resveratrol, mainly isolated from grape peel and other diversified edible plants such as *Passiflora edulis* Sims, *Vaccinium uliginosum* L., *Vitis vinifera* L., *Saccharum officinarum* L., etc. (Shrestha et al. 2019; Sochorova et al. 2020; Krambeck et al. 2020). Resveratrol and its analogs, including PIC, exhibit diverse pharmacological properties, such as antioxidant and anti‐inflammatory effects, and have been shown to mitigate thermally induced lipid oxidation (Liang et al. 2023). It is also an active component of various traditional Chinese medicines such as *Rheum palmatum* and *Polygonum cuspidatum* (Ha et al. 2018; Alperth et al. 2021). PIC demonstrates multiple pharmacological effects, including immunomodulatory, hypoglycemic, antibacterial, anti‐cancer, and anti‐α‐glucosylceramidase activities (Zheng et al. 2018; Wang et al. 2022; Dos Santos et al. 2022; Akinwumi et al. 2018; Shi et al. 2022; Alhakamy et al. 2020; Jiang et al. 2020). Its therapeutic potential depends on certain molecular processes, such as cell survival, enhanced cytotoxicity, autophagy activation, and signaling pathway regulation.

The present study systematically examined PIC as a potential dual‐action agent against *H. pylori* by targeting both bacterial growth and urease activity. The experimental approach included kinetic analysis of urease inhibition through activity assays and progress curve evaluation, identification of key active‐site interactions using thiol protection and metal competition experiments, and molecular docking to visualize binding modes.

## 2 Materials and Methods

### 2.1 Chemicals and Reagents

PIC (purity: ≥ 98%, CAS number: 10083‐24‐6) was supplied by Chengdu Desert Bio‐Technology Co. Ltd. (Chengdu, China). Acetohydroxamic acid (AHA, CAS number: 546‐88‐3) and Jack bean urease (JBU) (Type III, 40.3 U/mg) were offered by Sigma. Urea, dithiothreitol (DTT) and L‐cysteine (L‐cys) were provided by Solebo (Beijing, China). Glutathione (GSH) was supplied by Meilunbio Biologicals (Dalian, China). Sodium fluoride (NaF) and boric acid (BA) were offered by Macklin (Shanghai, China).

### 2.2 Preparation of *H. pylori* and Its Urease

The standard strain *H. pylori* strains (ATCC 43504) were cultured on Mueller‐Hinton agar supplemented with 5% sheep blood. Bacterial cultures were maintained for 72 h at 37°C in a microaerophilic environment (5% O_2_, 10% CO_2_, 85% N_2_). For urease extraction, bacterial cells were harvested by centrifugation (5000×*g*, 10 min, 4°C) and subsequently washed three times with phosphate‐buffered saline (pH 7.4). Following turbidity adjustment using McFarland standards, *H. pylori* urease was prepared via a method established by Matsubara et al. (2003).

### 2.3 Minimal Inhibitory Concentration Assay

The experimental procedure involved preparing Mueller‐Hinton agar medium according to standard protocols, with each 100 mL aliquot supplemented with PIC stock solution to achieve final concentrations of 0, 25, 50, 100, and 150 μg/mL. After solidification and overnight storage at 4°C to confirm sterility, plates showing no contamination were inoculated with 100 μL of *H. pylori* suspension (adjusted to McFarland 1.0 standard) and incubated microaerophilically (85% N_2_, 10% CO_2_, 5% O_2_) at 37°C for 5 days. The minimum inhibitory concentration (MIC) was then determined as the lowest drug concentration that completely inhibited visible bacterial growth, with all experiments performed in triplicate for each of the three *H. pylori* strains (ATCC 43504, HP1869, and HP1870) to ensure reproducibility.

### 2.4 Standard Urease Activity Assay

Urease activity was measured using spectrophotometry. The reaction was initiated by blending a particular amount of enzyme‐containing solution and 150 mM urea in 20 mM HEPES buffer (pH 7.5) at 37°C. Following 20 min, the Berthelot reagent was applied, and the quantity of ammonia produced by the urease‐urea reaction was detected at 595 nm (Wu et al. 2013). The control group represented the activity of the uninhibited enzyme, which is 100% active. One unit (U) of enzyme activity was defined as the amount of enzyme required to produce 1 μM ammonia per minute under these conditions. Three independent replicates were performed.

### 2.5 Urease Inhibition Test

Equal volumes of urease solution were mixed with a series of concentrations of PIC (0–2.0 mM) or AHA (0–0.2 mM) solution, and preincubated at 37°C for 20 min. Then 150 mM urea was added, and the reaction was carried out in the dark for 20 min. Afterward, the Berthelot reagent was added, and the quantity of ammonia produced by the urease‐urea reaction was detected at 595 nm. The residual enzyme activity (RA%) was calculated using the formula: RA% = activity with inhibitor/activity without inhibitor × 100%. The IC_50_ values were determined as the inhibitor concentration causing a 50% reduction in urease activity. Each experiment was repeated three times.

### 2.6 Determination of Urease Inhibition Type

The Lineweaver‐Burk plots were applied to calculate the change in the Michaelis constant (*K*_M_) and maximum velocity (*V*_max_). The *K*_M_ and *V*_max_ were assessed at different concentrations of PIC (0, 0.25, 0.50, 1.00 mM) by measuring the initial reaction rates across a range of urea concentrations (0.469–15 mM). Additionally, the inhibition constant was derived by secondary replotting of Lineweaver‐Burk plots. All tests were performed in triplicate.

### 2.7 Assessment of Inactivation Kinetics

Equal volumes of urea were reacted with a mixture of urease and different concentrations of PIC. Two approaches were compared: (1) PIC and urease were preincubated at 37°C for 20 min; (2) they were directly mixed without incubation. Enzyme activity in both procedures was measured as outlined in Section 2.4. Subsequently, the curve fitting software was used to fit the data according to the following formula:

$$P\left(t\right)=V_{S}\times t+\left(V_{0}-V_{S}\right)(1-e^{-\text{kapp}\times t)}/{\text{kapp}}^{-1}$$

where, *P*(*t*) is the amount of product accumulated after *t* minutes of reaction, *V*_0_ is the velocity at the start of the reaction, *V*_S_ is the velocity at which the reaction reaches equilibrium, and kapp is the apparent velocity constant (Breitenbach and Hausinger 1988). The experiments were repeated three times.

### 2.8 Protective Assay of PIC Suppressing Urease

#### 2.8.1 Protective Experiment of Sulfhydryl Compounds on Urease Inhibition

In this investigation, the effects of urease, PIC, and sulfhydryl compounds on urease activity were investigated. Firstly, the mixture containing urease, sulfhydryl compounds (1.25 mM DTT, GSH or L‐cys) and 1.5 mM PIC solution was incubated for 20 min at 37°C. Subsequently, the enzyme activity was evaluated as outlined in Section 2.4. The assay was performed in parallel three times.

#### 2.8.2 Protective Assay of Inorganic Reagent on Urease Inhibition

Firstly, the mixture was obtained by reacting the urease with 1.25 mM BA or NaF for 20 min at room temperature. Secondly, 1.5 mM PIC was co‐incubated with the mixture for 20 min. The enzyme activity was then assessed as described in Section 2.4. The trial was repeated three times in parallel.

### 2.9 PIC‐Thiol‐Urease Interplay Test

#### 2.9.1 Impact of Incubation Time on Urease Activity

In this experiment, urease, thiol‐containing substances (1.25 mM DTT, GSH, or L‐cys) and 1.5 mM PIC solution were mixed and co‐incubated at 37°C for 5, 10, 20, and 40 min. After that, the enzyme activity was assessed as described in Section 2.4. Each sample was tested in triplicate.

#### 2.9.2 Impact of Adding Order on Urease Activity

In this study, urease, thiol‐containing substances (1.25 mM DTT, GSH, or L‐cys), and 1.5 mM PIC solution were mixed in pairs. The effect of the addition order of the three solutions on urease activity was evaluated. The specific orders of addition were as follows: firstly, after 20 min of incubation of PIC with the thiol‐containing compounds, urease was added. Secondly, after 20 min of incubation of urease with the thiol‐containing compounds, PIC was added. Finally, after 20 min of incubation of urease with PIC, the thiol‐containing compounds were added.

### 2.10 Reactivation of PIC‐Deactivated Urease

The reactivation of PIC‐inactivated urease was inspected via the GSH method. Briefly, the urease was co‐incubated with an equal volume of PIC (1.5 mM) at 37°C. After 20 min, half of the reaction solution was removed and added to 1.25 mM GSH solution. Enzyme activity was determined before and after the addition of GSH. After co‐incubation for different periods, the residual enzyme activity in the mixtures was detected as described in Section 2.4.

### 2.11 Molecular Docking

To evaluate potential interactions between PIC and urease, molecular docking was performed using Autodock Vina (Xue et al. 2022). The crystal structures of HPU (PDB: 1E9Y, 3.00 Å resolution) and JBU (PDB: 3LA4, 2.05 Å resolution) were obtained from the RCSB Protein Data Bank. Proteins were prepared using PyMOL (removal of water molecules and addition of hydrogens), and both compounds and proteins were converted to pdbqt format using Autodock Tools 1.5.6. Blind docking was performed using 15 Å grid boxes with 0.375 Å spacing. The highest‐scoring conformations from Autodock Vina 1.1.2 were analyzed and visualized using PyMOL.

### 2.12 Statistical Analysis

Data were analyzed utilizing GraphPad Prism 8 (GraphPad Software Inc.) and SPSS 26.0 (SPSS Inc.). Data were presented using mean ± standard error (SEM). One‐way analysis of variance (ANOVA) was applied to the discrepancies between groups, and finally, Dunnett's test was performed. *p* < 0.05 was deemed statistically significant.

## 3 Results

### 3.1 MIC of PIC Against *H. pylori*

As demonstrated in Table 1 and Figure 1, PIC exhibited varying levels of inhibition on the growth of three *H. pylori* strains when cultured at a pH of 7.2. Notably, the MIC for PIC against the standard strain ATCC 43504 was determined to be 150 μg/mL. In contrast, PIC showed an MIC of 100 μg/mL for the clinical strains HP1869 and HP1870.

**TABLE 1:** Minimal inhibitory concentrations (MICs) of PIC against *Helicobacter pylori* strains.

Number	*Helicobacter pylori* strains	MIC (μg/mL)
1	ATCC 4304	150
2	HP1869	100
3	HP1870	100

### 3.2 Urease Inhibition Studies

**FIGURE 2:**
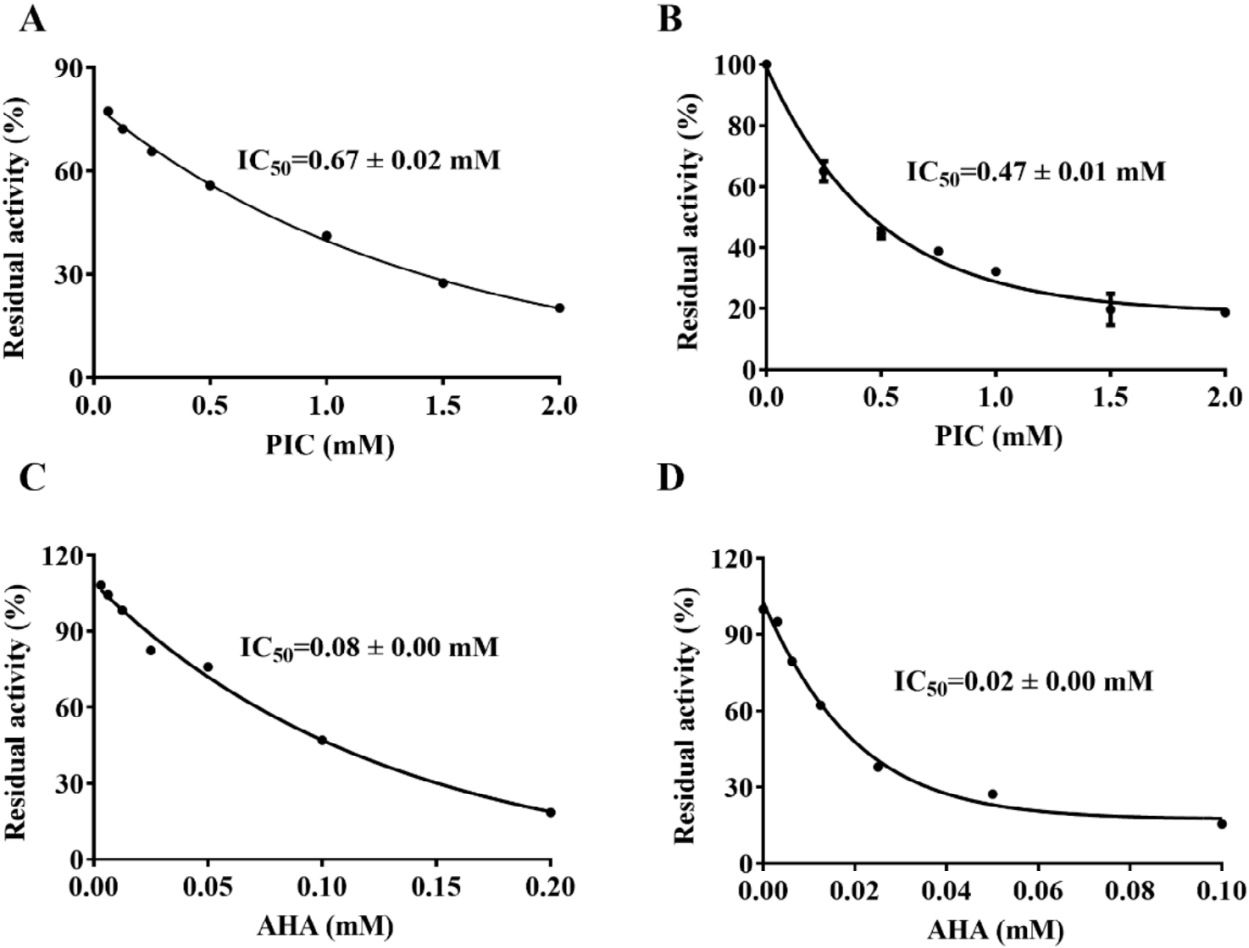
Suppressive role of PIC towards *Helicobacter pylori* urease (HPU) and jack bean urease (JBU) activity. Enzyme was treated using PIC (A: HPU, B: JBU) and AHA (C: HPU, D: JBU). The results presented are based on the averages obtained from three separate experimental trials.

As shown in Figure 2, PIC demonstrates a concentration‐dependent inhibitory effect on HPU and JBU, with the IC_50_ values of 0.67 ± 0.02 mM and 0.47 ± 0.01 mM, respectively. In comparison, the IC_50_ values for the positive control, AHA, are 0.08 ± 0.00 mM and 0.02 ± 0.00 mM, respectively.

### 3.3 Urease Inhibition Type Analysis

**FIGURE 3:**
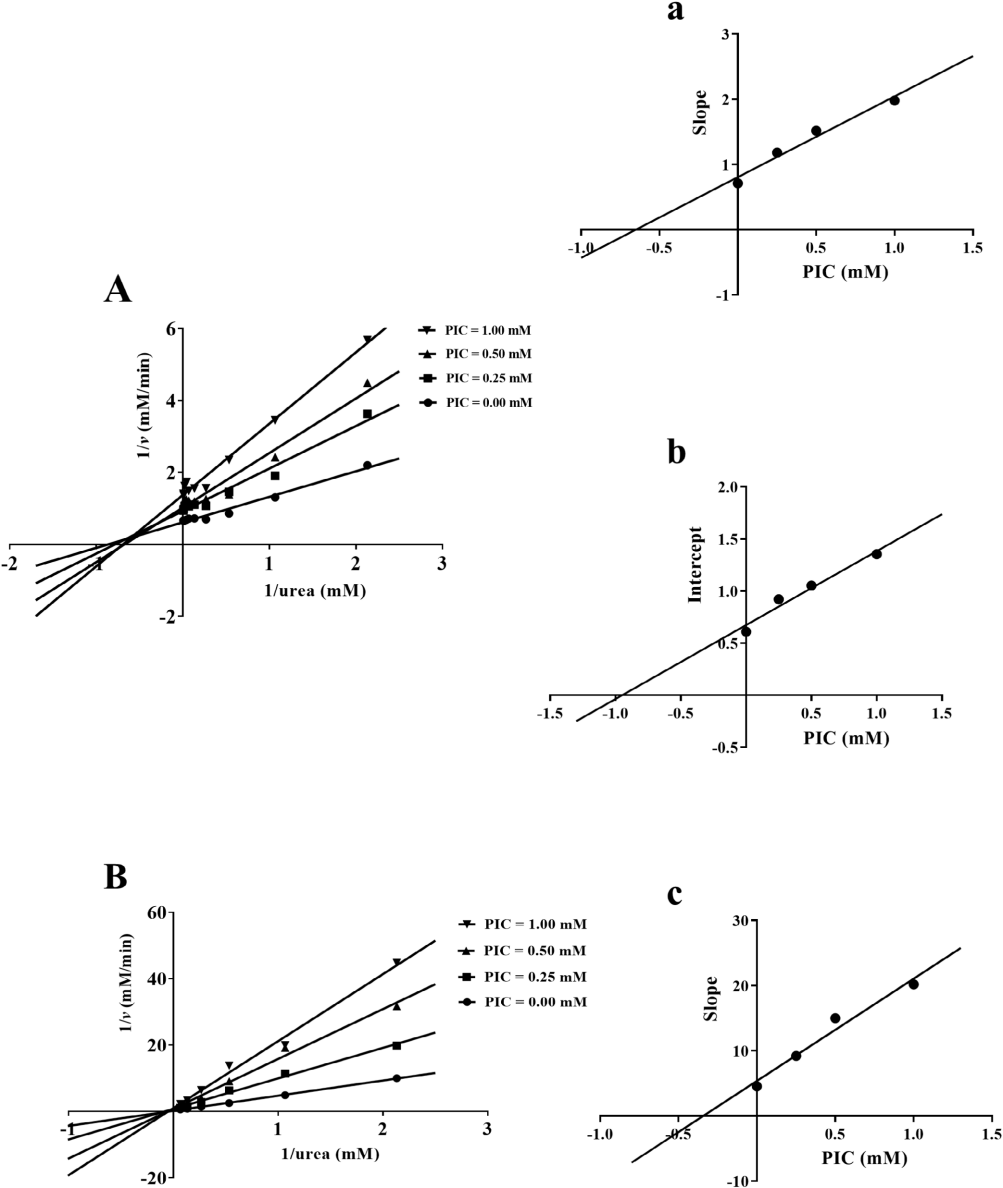
Kinetic analyses of urease suppression by PIC. Lineweaver‐Burk plots in the non‐existence and existence of varying PIC concentrations for HPU (A) and JBU (B). The secondary plots derived from the slopes of the Lineweaver‐Burk plots versus the different concentrations of PIC were created for HPU (a) and JBU (c). A secondary plot illustrating the relationship between the intercepts of the Lineweaver‐Burk plots and varying concentrations of PIC was generated for HPU (b). The test was performed three times.

As shown in Figure 3A, the Lineweaver‐Burk plot revealed that the kinetic parameter *K*_M_ for HPU inhibition by PIC increased with rising PIC concentrations, while *V*_max_ decreased. These results indicated that PIC acts as a mixed‐type inhibitor of HPU. Furthermore, the slope and intercept of the Lineweaver‐Burk plot varied with inhibitor concentration (Figure 3a,b). The inhibitory constants *Ki* and *Kis* were found to be 0.63 ± 0.02 mM and 0.94 ± 0.01 mM, respectively.

In addition, as illustrated in Figure 3B, with the variation in the concentration of PIC, the *K*_M_ value remained relatively constant, while the *V*_max_ value gradually decreased with increasing concentrations of PIC. This leads to an initial inference that the inhibitory effect of PIC on JBU is of the non‐competitive type. Additionally, a secondary plot of the Lineweaver‐Burk plot was created, and the inhibition constant *Ki* was determined to be 0.34 ± 0.05 mM (Figure 3c).

### 3.4 Reaction Progress Curves

**FIGURE 4:**
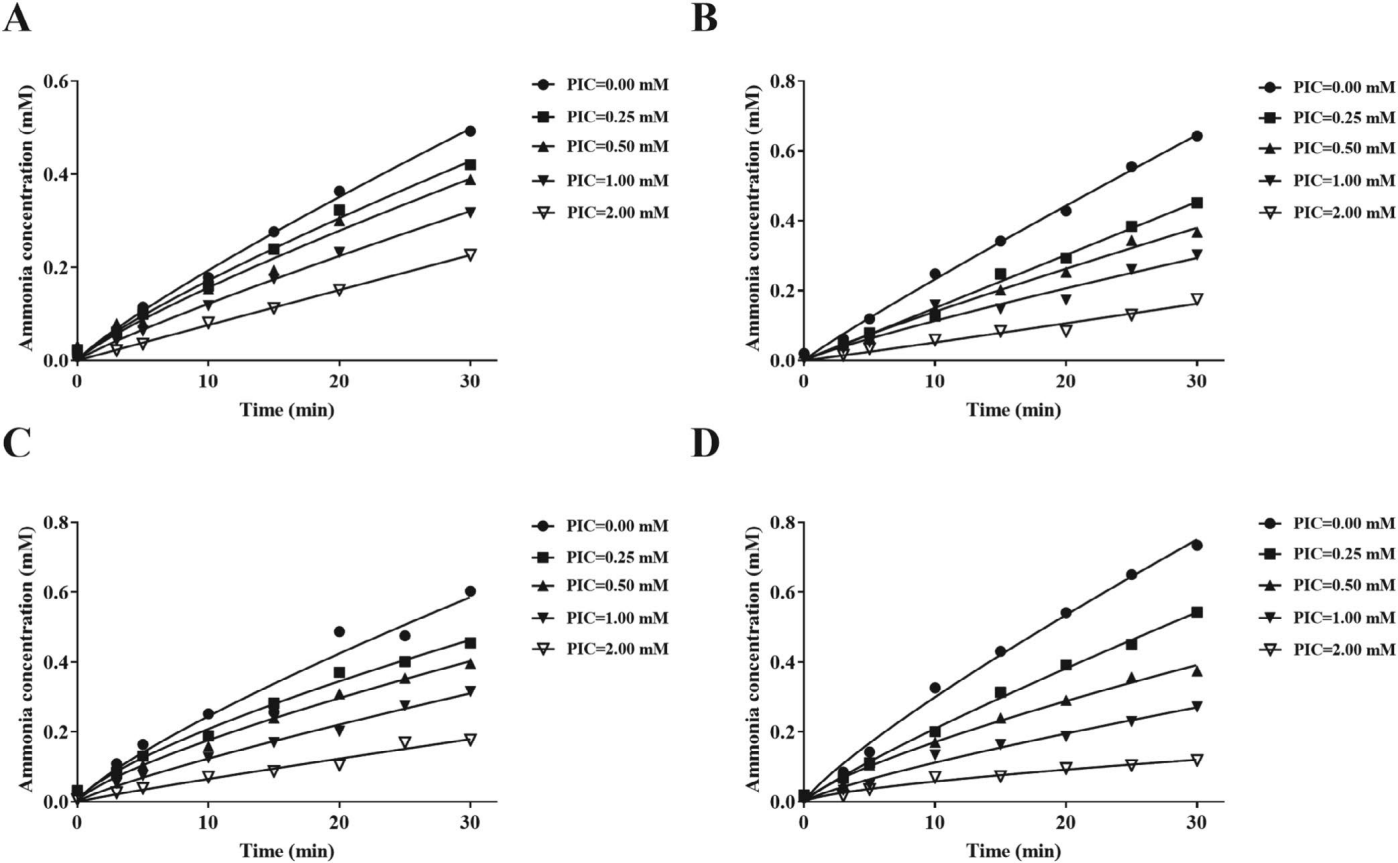
Reaction progress curves of urease‐catalyzed hydrolysis of urea in the non‐existence and existence of PIC. Reactive progress curves in the non‐coincubated for HPU (A) and JBU (C), and co‐incubated systems for HPU (B) and JBU (D). Curves were generated by examining the relationship between ammonia concentration and the duration of coincubation. The test was performed three separate times.

As indicated in Figure 4, PIC concentrations and co‐incubation time significantly affect the binding rate of urease‐PIC. Simultaneously, there is no noticeable discrepancy between the non‐incubated and the pre‐incubated system in the two groups for HPU and JBU. The inhibition process curve of HPU and JBU is characteristically convex upward, indicating that the binding was initiated by the rapid formation of a collision complex (EI) and followed by the gradual transition to a more stable final complex EI×. The rate of urea hydrolysis decreases from *V*_0_ to *V*_s_. The reaction progress curve of PIC on HPU and JBU aligns with the slow‐binding inhibition model described by Morrison and Walsh (1988).

### 3.5 Protective Experiment of PIC Repressing Urease

**FIGURE 5:**
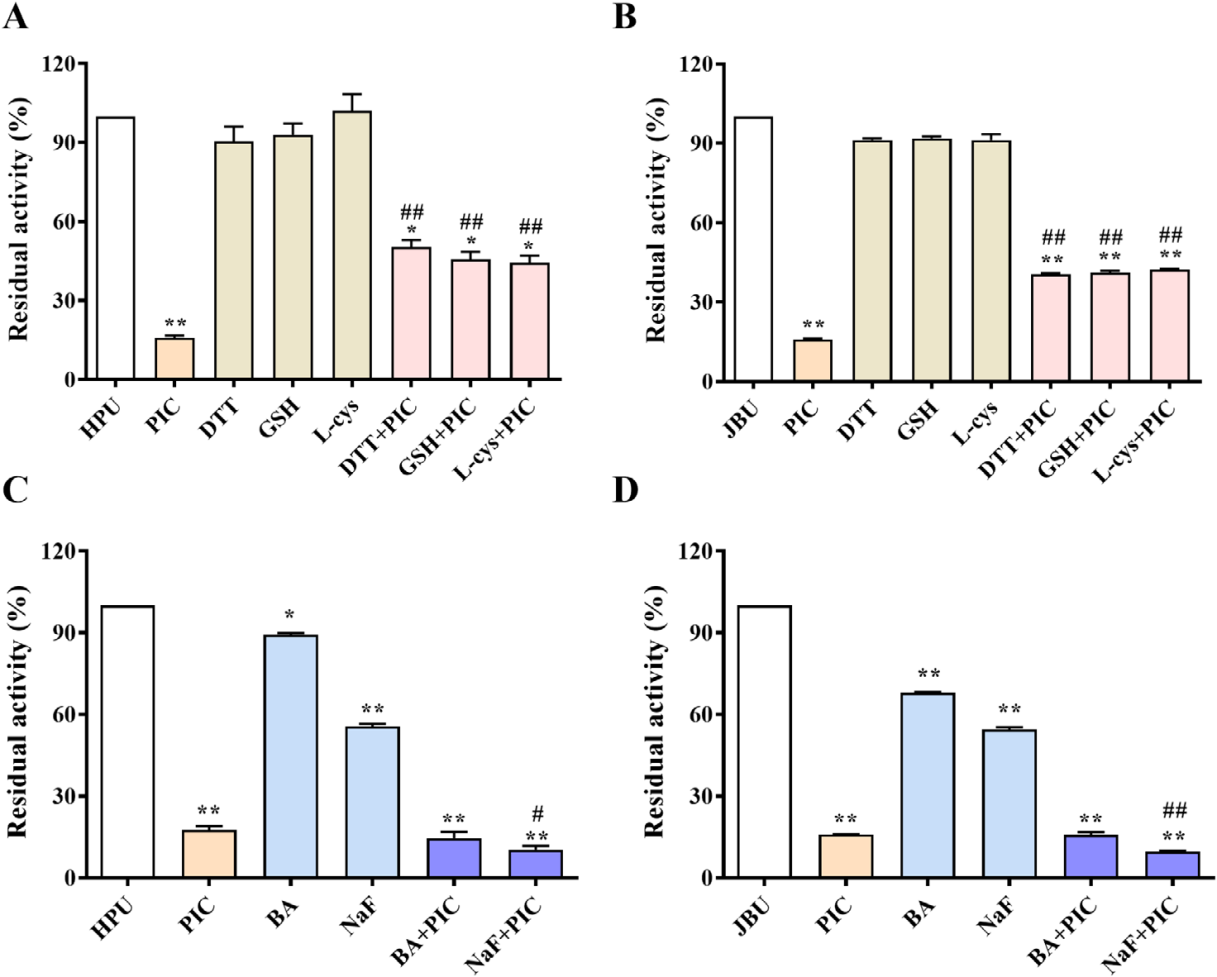
Suppressive properties of thiol‐containing substances and inorganic agents towards urease suppression by PIC. Influence of thiol‐containing agents (DTT, GSH, or L‐cys) towards HPU (A) and JBU (B) enzyme activity deactivation by PIC. Impact of inorganic substances (BA or NaF) towards HPU (C) and JBU (D) activity suppression by PIC. Data were presented using mean ± SEM from three separate assays. *p < 0.05, **p < 0.01 versus the urease group; ^#^ *p* < 0.05, ^##^ *p* < 0.01 versus the PIC group.

Studies have demonstrated that sulfhydryl compounds are recognized as activators of urease, as they are able to interact with the thiol groups (‐SH) in urease (Lu et al. 2021). Therefore, in this study, three thiol substances (DTT, GSH, L‐cys) were employed to probe the effect of PIC on the potential inactivation sites of urease. As manifested in Figure 5A,C, the activity of urease (including HPU and JBU) was significantly higher than that of PIC acting on urease alone when the reaction system contained thiol compounds. The results suggested that one of the action sites for the suppression of urease activity by PIC may be the thiol groups in the amino acid sequence of urease.

Inorganic substances (NaF and BA) react with the nickel ions (Ni^2+^) at the active site of urease and are generally employed as competitive inhibitors to study whether the target of inhibition by inactivators is the Ni^2+^ at the active site of urease (Mazzei, Cianci, Contaldo, and Ciurli 2019). Thus, in this study, NaF (a competitive slow‐binding inhibitor of urease) and BA (a typical competitive inhibitor of urease) were utilized to examine whether PIC interacts with the Ni^2+^. As indicated in Figure 5B,D, when both PIC and the inorganic compounds (NaF or BA) were present in the reaction system, the residual HPU and JBU activity was significantly lower than in the group where PIC acts alone. This suggests that inorganic compounds, particularly NaF, do not protect the vitality of urease and even work synergistically with PIC to inhibit its activity. The results also further implicate that the site of action for the suppression of HPU and JBU activity by PIC may be the thiol groups in the amino acid sequence of urease.

### 3.6 PIC‐Thiol‐Urease Interplay Test

**FIGURE 6:**
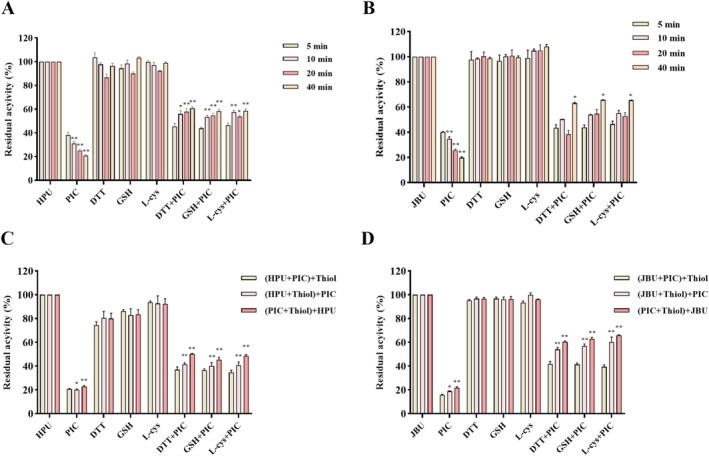
The protective role of incubation time and the sequence of addition of thiol compounds towards PIC‐modified HPU (A, C) and JBU (B, D). Enzymatic activity was examined following coincubation for 5, 10, 20, or 40 min. The constituents within the brackets were pre‐incubated for 20 min, after which the components outside the brackets were added for an additional 20 min of coincubation. The concentrations of PIC, thiol agents, and inorganic substances were 1.5, 1.25, and 1.25 mM, respectively. Data were presented using means ± SEM from three separate assays. *p < 0.05 and **p < 0.01 versus the first column of each group.

In the experiments with the addition of thiol compounds, the co‐incubation time possessed a certain influence on the enzyme activity. Thiol compounds (DTT, GSH and L‐cys) significantly protected the inhibitory effect of PIC on urease (including HPU and JBU) in a time‐dependent manner (Figure 6A,B). In addition, the enzyme activity was dependent on the addition order of PIC, urease, and thiol substances. Urease activity was greater when the sulfhydryl reagents were introduced initially compared to when the thiol compound was added following the co‐incubation of PIC and urease (Figure 6C,D). Taken together, these results fully support the key role of thiols in the deactivation of HPU and JBU by PIC.

### 3.7 Reactivation of PIC‐Deactivated Urease

**FIGURE 7:**
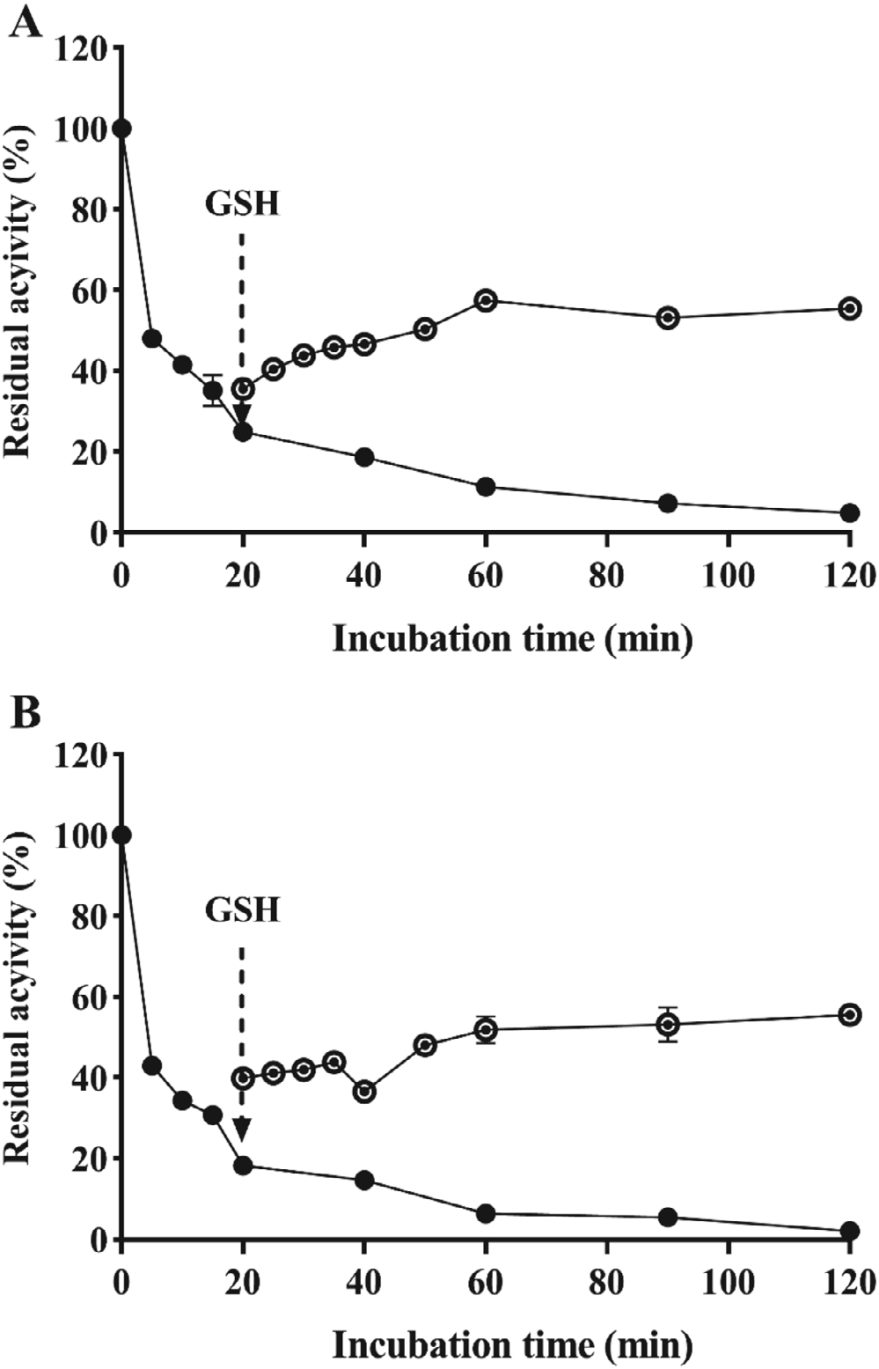
Enzymatic re‐activation of PIC‐evoked suppression of HPU (A) and JBU (B) by 1.25 mM GSH. The activity of urease was restrained by PIC (•) and subsequently assessed following the addition of GSH (◐). The test was performed in triplicate.

As shown in Figure 7, approximately 80% of the initial activity of HPU and JBU was lost after co‐incubation of urease with PIC for 20 min. However, the urease activity was restored to about 60% of the initial activity after adding 1.25 mM GSH. The findings suggested that the reaction between the enzyme and PIC is reversible. This result further supports the notion that PIC‐induced deactivation of HPU and JBU is associated with its binding to the thiol groups located at the active site of urease.

### 3.8 Molecular Docking Investigation

**FIGURE 8:**
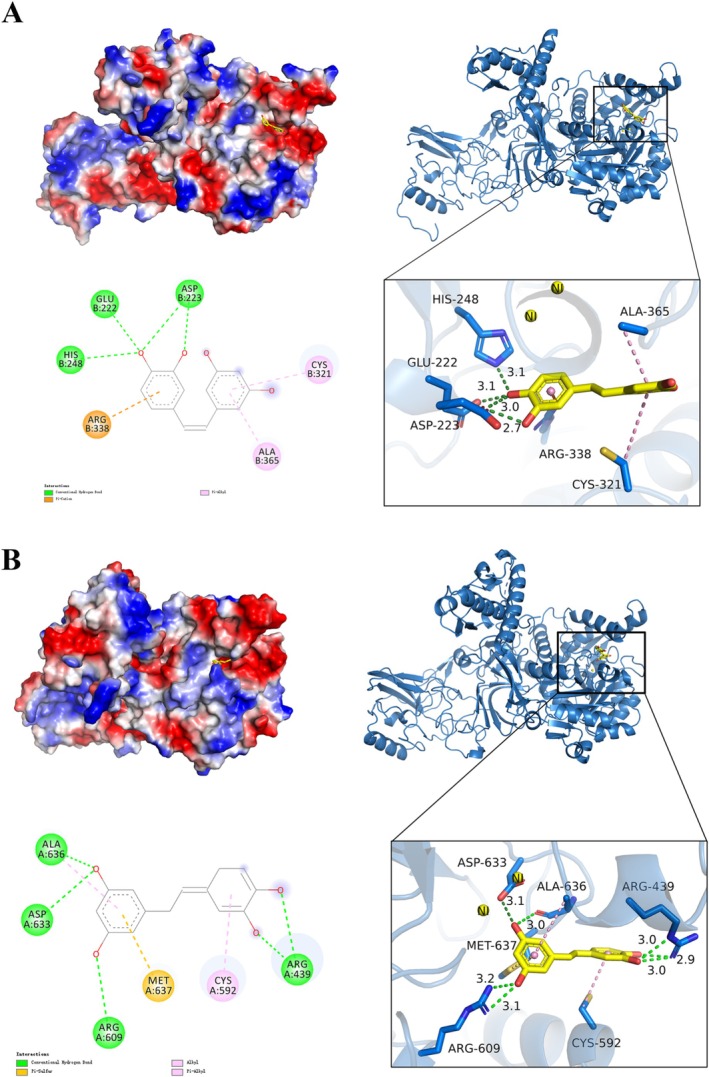
Molecular docking analysis of the interactions between PIC and HPU (A), and PIC and JBU (B). Schematic, three‐dimensional, and two‐dimensional representations depict the binding modes. Green, orange, and pink dashed lines correspond to hydrogen bonds, π–sulfur, and π–alkyl interactions, respectively.

As shown in Figure 8, PIC can effectively bind to the active pocket of HPU and JBU, exhibiting binding energies of −6.4 kcal/mol and −7.0 kcal/mol, respectively. Interaction analysis revealed that PIC anchors to the helix‐turn‐helix motif within the functional cavity, preventing the flap from returning to its closed conformation. Specifically, for HPU, PIC forms hydrogen bonds with amino acid residues HIS‐248 (3.1 Å), GLU‐222 (3.1 Å), and ASP‐223 (3.0 Å, 2.7 Å), respectively. The benzene ring of PIC formed π‐alkyl interactions with proteins ALA‐365 and CYS‐321, and formed π‐cation interactions with ARG‐388. For JBU, PIC formed hydrogen bonds with ARG‐439 (2.9 Å, 3.0 Å, 3.0 Å), ARG‐609 (3.1 Å, 3.2 Å), ASP‐633 (3.1 Å), and ALA‐636 (3.0 Å), respectively. The benzene ring of PIC established π‐alkyl bonds with protein residues ALA‐636 and CYS‐592, and formed π‐sulfur interactions with MET‐637.

## 4 Discussion

*H. pylori* is among the most common infectious pathogens affecting the gastrointestinal tract. Its colonization of the stomach leads to chronic inflammation of the gastric mucosa, which can progress to more serious gastric conditions (Wang, Yun, et al. 2024). As the use of antimicrobials to treat *H. pylori* infections rises, several challenges arise, such as declining cure rates, adverse drug reactions, and increased antibiotic resistance (Benito et al. 2024; Liu and Nahata 2023). PIC, a polyphenolic compound, is mainly found in grape skin, passionfruit, and red wine, and is also an active component in various traditional Chinese medicines such as *Rheum palmatum* and *Polygonum cuspidatum* (Afzali et al. 2019; Kunsorn et al. 2024; Banik et al. 2020; Benová et al. 2008). PIC has been reported to have various pharmacological effects, including anti‐bacteria, anti‐oxidation, immune enhancement, hypoglycemia, anti‐tumor, and anti‐inflammation (Cao et al. 2020; Gandhi et al. 2024). Similar to quercetin, which attenuates organ injury via antioxidant pathways (Cheng et al. 2024; Zhang et al. 2024; Zeng et al. 2023), PIC's suppression of *H. pylori* urease may involve scavenging reactive species generated during bacterial colonization. In our study, we observed that PIC showed inhibitory effects against three *H. pylori* strains: ATCC 43504, HP1869, and HP1870, with MIC values ranging from 100 to 150 μg/mL.

Urease is a key target for drug research in the fields of pharmaceuticals and agriculture. Focusing on urease may provide innovative strategies for dealing with conditions stemming from urease‐producing bacteria, such as *H. pylori* . *H. pylori* urease (HPU) is an important colonizing and pathogenic factor for *H. pylori* (Graham and Miftahussurur 2018; Cunha et al. 2021). Recent studies suggest that microbial metabolites, such as butyrate, can modulate host‐pathogen interactions via enzymatic lactylation (Wang et al. 2025). Similarly, PIC's suppression of urease activity may extend beyond direct inhibition to epigenetic or post‐translational regulation of bacterial virulence. In vivo, HPU degrades urea to NH_3_, CO_2_, and HCO_3_^−^. The generated NH_3_ neutralizes gastric acid locally, creating a pH‐buffered microhabitat that enables *H. pylori* survival in the highly acidic stomach, while simultaneously damaging gastric epithelial tissue and triggering inflammation (Souza et al. 2019; Wu et al. 2024). This dual role makes urease inhibition a strategic therapeutic target against *H. pylori* infections. In agriculture, urease is a vital target for the development of nitrogen sources, as it plays a significant role in the hydrolysis of urea into ammonia and carbon dioxide, influencing nitrogen availability for plants (Cantarella et al. 2018). The rapid decomposition of urea by urease not only causes the waste of agricultural resources but also triggers a range of ecological and environmental problems, including the release of ammonia gas, soil acidification, soil compaction, the “burning seedling” phenomenon in plants, and the pollution of nitrates in agricultural products and groundwater (Adetunji et al. 2017; Kafarski and Talma 2018; Modolo et al. 2015). Therefore, inhibiting urease can help reduce gastrointestinal diseases associated with *H. pylori* , enhance nitrogen use efficiency in agricultural practices, and minimize the environmental impacts related to nitrogen fertilizers. This dual benefit underscores the importance of urease inhibitors in both medical and agricultural contexts.

In earlier tests, diverse natural products, especially polyphenolic compounds, have been proven to effectively inhibit urease activity. Among them, Xiao et al. (2007) synthesized 20 polyphenolic compounds and found that 7,8,4′‐trihydroxyisoflavone (IC_50_ = 0.14 mM) exhibited potency in inhibiting HPU (Xiao et al. 2007). Wu et al. (2013) found that scutellarin exhibited strong inhibitory effects on JBU, with an IC_50_ value of 1.35 ± 0.15 mM (Wu et al. 2013). Baicalin was discovered to show inhibitory effects on HPU and JBU, with IC_50_ values of 0.82 ± 0.07 mM and 2.74 ± 0.51 mM, respectively (Tan et al. 2013; Yu et al. 2015). Consistent with previous reports, our study revealed that PIC exhibited significant inhibitory effects on HPU and JBU, with an IC_50_ of 0.63 ± 0.02 mM and 0.47 ± 0.01 mM, respectively. These findings provide valuable clues for the development of novel urease inactivators. The superior urease inhibitory activity of PIC compared to these reference compounds, combined with its established safety profile, positions it as a promising candidate for both therapeutic development against *H. pylori* infections and as a biodegradable urease inhibitor for sustainable agriculture applications.

Ureases from various sources contain distinct types of subunits. Nonetheless, they all have more than 50% similarity to one another, which indicates that they share common catalytic properties. This structural conservation allows for similar mechanisms of action across different ureases, despite variations in their subunit composition. JBU, the first crystallized and most well‐characterized urease, is extensively utilized as a model enzyme for inhibition assays (Sumner 1926). JBU is a homo‐hexamer that possesses two Ni^2+^ and 15 cysteine residues in each subunit (~91 kDa). By comparison, HPU is formed by two subunits: an α subunit (61.7 kDa) and a β subunit (26.5 kDa). The α subunit features a short homologous polypeptide chain related to the N‐terminal domain of JBU, while the β subunit contains a longer chain that includes the catalytic site (Follmer 2008). In the present study, kinetic analysis indicated that the repressive mode of PIC is mixed on HPU, whereas it is non‐competitive on JBU. The differences in kinetic activity are probably due to the different origins and structures of HPU and JBU. These distinct inhibition patterns actually enhance PIC's versatility, with mixed‐type inhibition being advantageous for clinical applications where complete urease blockade is desired, while non‐competitive inhibition benefits agricultural use by providing more consistent activity across varying soil pH conditions.

The change curve of ammonia production with incubation time can clearly reflect the dynamic progress of the enzymatic reaction (Lu et al. 2022; Silva et al. 2017). In this study, two different methods were used to validate the reaction process: one is the direct addition of urea to start the reaction without coincubation of urease and PIC; the other is the pre‐incubation of urease and PIC at 37°C, followed by the addition of urea to initiate the reaction. The experimental results showed that the enzymic inhibitor I (EI) complex progressively accumulated and eventually transformed into the EI× complex (E + I → EI → EI×). According to Morrison and Walsh's (1988) inhibition theory, this characteristic transformation process is considered to be slow binding inhibition (Morrison and Walsh 1988). This slow‐binding characteristic is particularly valuable for both medical and agricultural applications, enabling sustained urease inhibition in the gastric environment between oral doses in clinical use, while providing extended nitrogen retention in soil applications.

Within the structural unit of urease, Ni^2+^ and thiols, particularly the cysteine residues located at the active site of the enzyme, are widely recognized as critical components for the catalytic activity of urease (Montazer et al. 2024; Xie et al. 2019). Thiol‐containing substances such as DTT, L‐cys, and GSH effectively inhibit urease activity via interacting with the thiols of the enzyme (Krajewska and Zaborska 2007). Inorganic compounds (NaF and BA) exert their inhibitory effect by binding to the nickel ions at the active site of urease (Mazzei, Cianci, Contaldo, and Ciurli 2019). The results of this study demonstrate that thiol reagents (DTT, GSH, and L‐cys) have a strong protective effect on the inhibition of PIC on HPU and JBU, with GSH showing a particularly significant effect on the restoration of urease inhibition, indicating that the active site thiols may be the primary target for PIC inhibitory action on HPU and JBU. Several studies have also established that the thiols in urease are the primary target for enzyme inhibitors and that thiol reagents can partially restore their inhibitory effect (Lu et al. 2025; Mazzei et al. 2021; Tan et al. 2017). The inactivation of HPU and JBU caused by PIC was reversible, suggesting a favorable safety profile. As demonstrated in comprehensive reviews of medicinal plants like *Ruta graveolens* (Luo et al. 2024), thorough toxicological evaluation remains essential when developing plant‐derived therapeutics, even for compounds with established traditional use. In addition, incubation time and the addition order of the components significantly affect the inactivation of HPU and JBU by PIC. Coincubation of BA or NaF with PIC can induce more pronounced inhibitory effects, suggesting the possibility of a synergistic inhibitory role between PIC and NaF or BA. The recovery of PIC‐modified HPU and JBU activity by GSH and the protective effect of thiol‐containing substances further support the key mechanism of urease inactivation involving the binding of PIC to the active site thiols of urease.

Molecular docking approach is widely used to reveal the interactions between ligand compounds and receptor target protein. In this study, the Auto‐docking Vina software was applied to probe the potential binding mode of PIC with urease, in order to further validate the interaction sites between PIC and urease including HPU and JBU. The results revealed that PIC forms strong hydrogen bonds, π‐cation, and π‐alkyl interactions with several amino acid residues including HIS‐248, GLU‐222, ASP‐223, ALA‐365, CYS‐321, and ARG‐388 in the flap region of the active center on HPU. By comparison, PIC forms hydrogen bonds, π‐sulfur, and π‐alkyl interactions with several amino acid residues including ARG‐439, ARG‐609, ASP‐633, ALA‐636, MET‐637, and CYS‐592, which bind to JBU. These interactions keep the flap conformation in an open state, thereby reducing enzyme activity. Therefore, the molecular docking results further support the hypothesis that thiol‐containing residues at the active site may play a critical role in PIC‐mediated urease inactivation.

This study confirms the strong anti‐urease activity of PIC, but several limitations should be noted. First, all experiments were conducted in vitro; future studies should validate these findings in animal models of *H. pylori* infection. Second, the potential interactions between PIC and standard antibiotics (e.g., clarithromycin or metronidazole) remain unexplored. Third, the exact pharmacokinetic profile of PIC in the gastric environment requires further investigation. We propose three key research directions: (1) in vivo efficacy testing using *H. pylori* ‐infected murine models, (2) combination therapy studies with current antibiotics, and (3) formulation development to enhance PIC's gastric retention and stability. These investigations will better elucidate PIC's translational potential as an anti‐ *H. pylori* agent.

## 5 Conclusion

**FIGURE 9:**
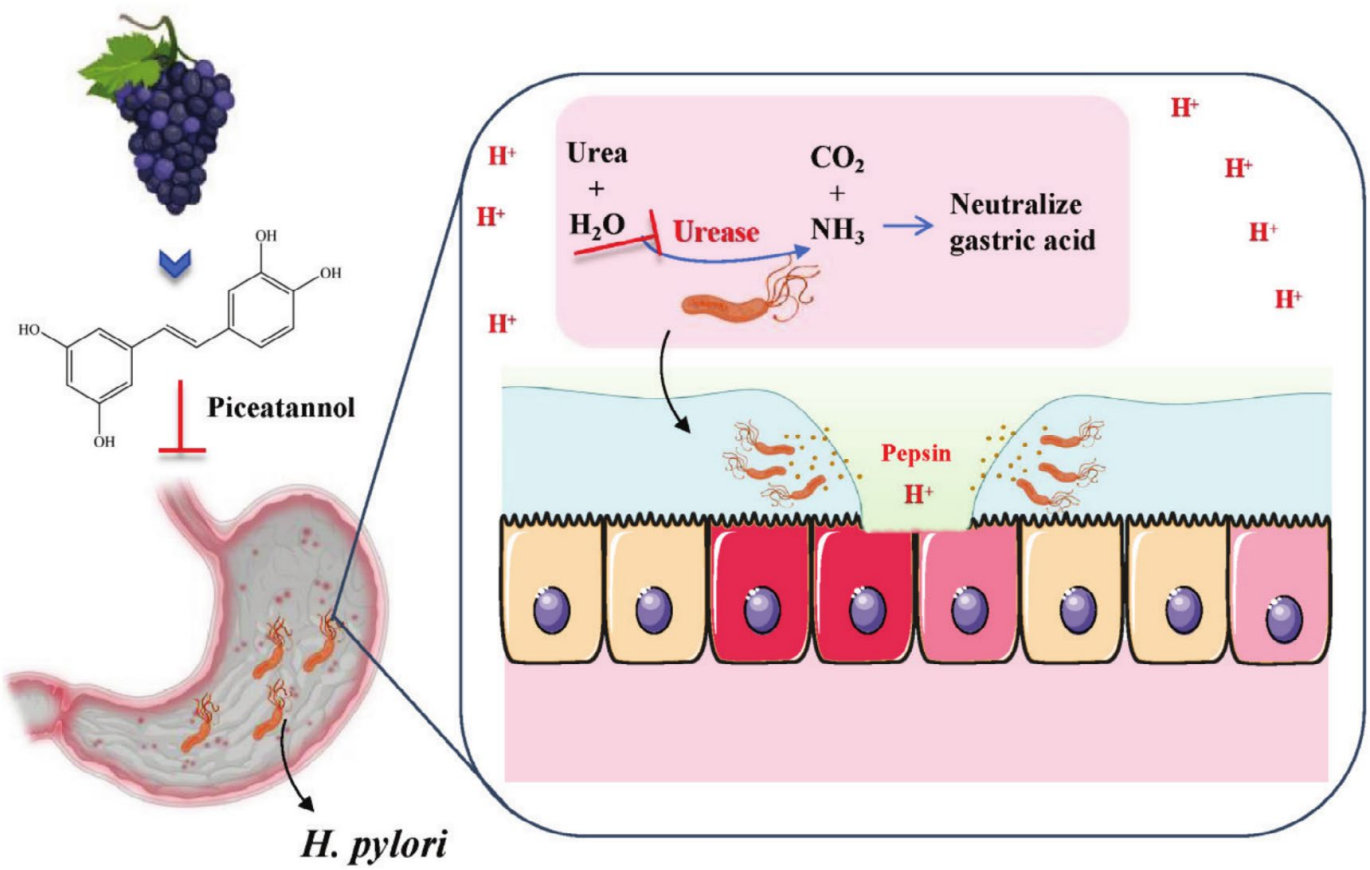
PIC, primarily extracted from grapes, effectively suppresses the growth of *Helicobacter pylori* through inhibiting its secreted urease activity. This inhibitory effect highlights its potential applications in medicine for the treatment of gastrointestinal diseases associated with *H. pylori* infection.

In summary, the findings of this study demonstrated that PIC significantly hindered the growth of *H. pylori* , at least in part, by suppressing its virulence factor, urease (Figure 9). Further enzyme kinetics, protective assays, and reactivation experiments have shown that PIC can bind to the active center thiols of the HPU and JBU in a concentration‐time‐dependent, slow‐binding, and reversible pattern. These characteristics make PIC a highly promising natural urease inhibitor. The findings provide a scientific basis for exploring the use of polyphenolic compounds like PIC in the treatment of gastrointestinal diseases induced by *H. pylori* . Additionally, they suggest potential applications in agriculture to improve soil nitrogen utilization efficiency. Overall, PIC represents a promising candidate for further research and development in both medicinal and agricultural contexts.

## Author Contributions

**Qiang Lu:** conceptualization (equal), investigation (equal), methodology (equal), writing – original draft (equal). **Yuhong Xie:** investigation (equal), writing – original draft (equal). **Xuefei Wang:** data curation (equal), investigation (equal). **Haohui Chen:** formal analysis (equal), investigation (equal). **Yifei Xu:** conceptualization (equal), methodology (equal), resources (equal), writing – review and editing (equal). **Cailan Li:** conceptualization (equal), funding acquisition (equal), project administration (equal), supervision (equal), writing – review and editing (equal).

## Ethics Statement

The authors have nothing to report.

## Conflicts of Interest

The authors declare no conflicts of interest.

## Data Availability

Data available on request from the corresponding author.
